# Maternal Exposure to the Cannabinoid Agonist WIN 55,12,2 during Lactation Induces Lasting Behavioral and Synaptic Alterations in the Rat Adult Offspring of Both Sexes

**DOI:** 10.1523/ENEURO.0144-20.2020

**Published:** 2020-09-15

**Authors:** Andrew F. Scheyer, Milene Borsoi, Anne-Laure Pelissier-Alicot, Olivier J.J. Manzoni

**Affiliations:** 1 Institut de Neurobiologie de la Méditerranée, Institut National de la Santé et de la Recherche Médicale Unité 1249, Marseille, France; 2 Aix-Marseille University, France, 13273; 3 Cannalab Cannabinoids Neuroscience Research International Associated Laboratory, Institut National de la Santé et de la Recherche Médicale-Aix-Marseille University/Indiana University, 13273; 4 Service de Psychiatrie, Assistance Public Hopitaux de Marseille, Centre Hospitalier Universitaire Conception, Marseille 13005, France; 5 Service de Médecine Légale, APHM, CHU Timone Adultes, Marseille 13005, France

**Keywords:** accumbens, cannabinoid, lactation, perinatal, prefrontal cortex

## Abstract

Consumption of cannabis during pregnancy and the lactation period is a rising public health concern (Scheyer et al., 2019). Exposure to synthetic or plant-derived cannabinoids via lactation disrupts the development of GABAergic neurons in the prefrontal cortex (PFC) and alters early-life behaviors (Scheyer et al., 2020b). Recently, additional data revealed that Δ9-tetrahydrocannabinol (THC) perinatal exposure via lactation causes lasting behavioral and neuronal consequences (Scheyer et al., 2020a). Here, the long-term effects in adult offspring of maternal exposure to the synthetic cannabinoid agonist WIN 55,12,2 are reported. The data demonstrate that rats exposed during lactation to WIN display social and motivational deficits at adulthood. These behavioral changes were paralleled by a specific loss of endocannabinoid-mediated long-term depression (eCB-LTD) in the PFC and nucleus accumbens (NAc), while other forms of synaptic plasticity remained intact. Thus, similarly to THC, perinatal WIN exposure via lactation induces behavioral and synaptic abnormalities lasting into adulthood.

## Significance Statement

Consumption of cannabis during pregnancy and the lactation period is a rising public health concern. Exposure to synthetic or plant-derived cannabinoids via lactation disrupts perinatal programming in the prefrontal cortex (PFC) and early-life behaviors. Here, we explored the long-term effects of maternal exposure to the synthetic cannabinoid agonist WIN 55,12,2 in the adult offspring. The results indicate that rats exposed during lactation to WIN display social and motivational deficits at adulthood. These behavioral changes were paralleled by a specific loss of endocannabinoid-mediated long-term depression (eCB-LTD) in the PFC and nucleus accumbens (NAc), while other forms of synaptic plasticity remained intact.

## Introduction

Cannabis consumption by pregnant women is progressively increasing (Hurd et al., 2019; Scheyer et al., 2019). The principle psychoactive component of cannabis, Δ9-tetrahydrocannabinol (THC), in addition to other cannabinoids, is actively transferred to the developing infant via breastfeeding (Hurd et al., 2019; Scheyer et al., 2019). During the perinatal period (i.e., during prenatal and early postnatal development), the developing brain is acutely sensitive to exogenous cannabinoids (Scheyer et al., 2019). Exposure to cannabinoids via lactation alters the developmental trajectory of the prefrontal cortex (PFC), which has been identified as a cortical hub essential to planning, cognitive flexibility, and emotional behaviors (Goldman-Rakic, 1991) and a common target in various endocannabinoid (eCB)-related synaptopathies (Araque et al., 2017). Thus, exposure of lactating females to either THC, or a synthetic agonist of CB1R, altered the maturational trajectory of GABAergic transmission and led to behavioral abnormalities in early life (Scheyer et al., 2020b). Disruptions to GABAergic development are known to occur following adolescent exposure to THC, as well (Renard et al., 2017). Furthermore, perinatal THC exposure via lactation elicits lasting, deleterious impacts on social behavior and synaptic plasticity in the PFC of the adult offspring (Scheyer et al., 2020a).

Here, we investigated the effects in both sexes of adult offspring of maternal exposure to the synthetic cannabinoid agonist, WIN 55,12,2 (WIN). These data demonstrate that rats exposed during lactation to WIN display social and motivational deficits at adulthood. These behavioral changes were paralleled by a specific loss of eCB-mediated long-term depression (eCB-LTD) in the PFC and nucleus accumbens (NAc), while other forms of synaptic plasticity remained intact. Thus, perinatal WIN exposure via lactation induces behavior and synaptic abnormalities lasting into adulthood.

## Materials and Methods

### Animals

Animals were treated in compliance with the European Communities Council Directive (86/609/EEC) and the United States National Institutes of Health Guide for the Care and Use of Laboratory Animals. All animal procedures were performed in accordance with the Aix-Marseille University and INSERM animal care committee’s regulations. All rats were group-housed with 12/12 h light/dark cycles with *ad libitum* access to food and water [zeitgeber time (ZT)0 = 7 A.M.]. All behavioral, biochemical and synaptic plasticity experiments were performed on male and female RjHan:wi-Wistar rats (> post-natal day (P)90) from pregnant females obtained from Janvier Labs. Pregnant dams arrived at embryonic day (E15) and remained undisturbed until delivery. Newborn litters found before 5 P.M. were considered to be born that day (P0). Dams were injected daily subcutaneously from P01 to P10 with WIN (0.5 mg/kg/d), dissolved in 10% polyethylene glycol/10% Tween/80% saline and injected subcutaneously (Borsoi et al., 2019). Control dams (sham) received vehicle.

### Behavioral procedures

#### Open field

Observations were conducted after rats were adapted to the room laboratory conditions for at least 1 h before testing. Tests were conducted in a 45 × 45 cm transparent Plexiglas arena. All behavioral procedures were performed between 10 A.M. and 3 P.M. A video tracking system (Ethovision XT, Noldus Information Technology) recorded the total distance traveled and time spent in the central zone (21 × 21 cm) of the apparatus (Borsoi et al., 2019).

#### Social interaction

The apparatus consisted of a transparent acrylic chamber (120 × 80 cm) divided into three equal compartments (40 cm each) partially separated by white walls. The central compartment was empty and lateral compartments had an empty wire cage (20 cm in diameter) were an object or a new rat (social stimulus) were placed during the test. WIN or sham-exposed rats were individually habituated to the test cage containing the two empty wire cages for 5 min immediately before testing. The first trial (social approach, 5-min duration) consisted of giving the tested rat the option to socialize with either a novel object or a new, naive, age-mate, and sex-mate conspecific rat that were placed into the wire cages positioned on the arena’s opposite sides; 30 min later, the tested rat returned to the apparatus for the second trial (social memory, 5-min duration) wherein the two compartments held either the now-familiar rat from the first testing phase or a second, previously unknown, naive, age-mate, and sex-mate conspecific.

Only rats with no compartment preference during the habituation phase were used. Time spent in each compartment and time spent exploring wire cages during the social approach and social memory phases were scored. Social preference ratio was calculated as time spent exploring either the wire cage containing the object, or the new rat divided by total time exploring both wire cages. Likewise, social memory ratio was calculated as time spent exploring either the wire cage containing the rat used in the first trial or the new rat divided by total time exploring both wire cages. Recognition index >0.5 indicates preferable object recognition memory.

#### Anhedonia

We performed sucrose consumption tests (Monleon et al., 1995; Bessa et al., 2009). Rats were exposed for 24 h to a bottle containing a sucrose solution (5% in tap water, Sigma), placed in the wire-top cage cover adjacent to standard tap water, followed by 12 h of water deprivation and a 20-min exposure to two identical bottles (one filled with 5% sucrose solution and the other with water). Bottles were placed at opposite ends of the cage and counterbalanced across groups to avoid side bias. Sucrose preference was calculated as the ratio of the volume of sucrose versus volume or consumed during the 20-min test. All animals were habituated to the testing room 24 h before initiating the sucrose preference test.

#### Slice preparation

Adult male and female rats were anesthetized with isoflurane and killed (Bara et al., 2018; Borsoi et al., 2019). The brain was sliced (300 μm) in the coronal plane with a vibratome (Integraslice, Campden Instruments) in a sucrose-based solution at 4°C (87 mm NaCl, 75 mm sucrose, 25 mm glucose, 2.5 mm KCl, 4 mm MgCl_2_, 0.5 mm CaCl_2_, 23 mm NaHCO_3_, and 1.25 mm NaH_2_PO_4_). Immediately after cutting, slices containing the medial PFC or the NAc were stored for 1 h at 32°C in a low-calcium artificial CSF (ACSF) that contained the following: 130 mm NaCl, 11 mm glucose, 2.5 mm KCl, 2.4 mm MgCl_2_, 1.2 mm CaCl_2_, 23 mm NaHCO_3_, and 1.2 mm NaH_2_PO_4_, and were equilibrated with 95% O_2_/5% CO_2_ and then at room temperature until the time of recording. During the recording, slices were placed in the recording chamber and superfused at 2 ml/min with low Ca^2+^ or normal Ca^2+^ ACSF (PFC and NAc, respectively). All experiments were done at 32°C (PFC) or 25°C (NAc). The superfusion medium contained picrotoxin (100 mm) to block GABA-A receptors. All drugs were added at the final concentration to the superfusion medium.

### Electrophysiology

Whole-cell patch clamp of visualized layer five pyramidal medial PFC or medium spiny neurons (MSNs) and field potential recordings were made in coronal slices using standard procedures (Bara et al., 2018; Borsoi et al., 2019). Neurons were visualized using an upright microscope with infrared illumination. The intracellular solution was based on K^+^ gluconate (145 mm K^+^ gluconate, 3 mm NaCl, 1 mm MgCl_2_, 1 mm EGTA, 0.3 mm CaCl_2_, 2 mm Na^2+^ ATP, 0.3 mm Na^+^ GTP, and 0.2 mm cAMP, buffered with 10 HEPES). The pH was adjusted to 7.2 and osmolarity to 290–300 mOsm. Electrode resistance was 4–6 MΩ. Recordings were performed with an Axopatch-200B amplifier. Data were low pass filtered at 2 kHz, digitized (10 kHz, DigiData 1440A, Axon Instrument), collected using Clampex 10.2, and analyzed using Clampfit 10.2 (all from Molecular Device).

A −2-mV hyperpolarizing pulse was applied before each evoked EPSC to evaluate the access resistance and those experiments in which this parameter changed >25% were rejected. Access resistance compensation was not used, and acceptable access resistance was <30 MΩ. The potential reference of the amplifier was adjusted to zero before breaking into the cell. Cells were held at –75 mV.

Current-voltage (I-V) curves were made by a series of hyperpolarizing to depolarizing current steps immediately after breaking into the cell. Membrane resistance was estimated from the I-V curve around resting membrane potential (Martin et al., 2015). Field potential recordings were made in coronal slices containing the PFC or the NAc (Kasanetz et al., 2013). During the recording, slices were placed in the recording chamber and superfused at 2 ml/min with low Ca^2+^ ACSF. All experiments were done at 32°C. The superfusion medium contained picrotoxin (100 mm) to block GABA-A receptors. All drugs were added at the final concentration to the superfusion medium. The glutamatergic nature of the field EPSP (fEPSP) was systematically confirmed at the end of the experiments using the ionotropic glutamate receptor antagonist CNQX (20 mm), which specifically blocked the synaptic component without altering the non-synaptic.

Both fEPSP area and amplitude were analyzed. Stimulation was performed with a glass electrode filled with ACSF and the stimulus intensity was adjusted ∼60% of maximal intensity after performing an input–output curve (baseline EPSC amplitudes ranged between 50 and 150 pA). Stimulation frequency was set at 0.1 Hz.

### Data acquisition and analysis

The magnitude of plasticity was calculated at 0–10 and 30–40 min after induction (for Theta Burst Stimulation (TBS)-LTP and eCB-LTD) or drug application (mGlu2/3-LTD) as percentage of baseline responses. Statistical analysis of data was performed with Prism (GraphPad Software) using tests indicated in the main text after outlier subtraction (Grubb’s test, α level 0.05). All values are given as mean ±SEM, and statistical significance was set at *p* < 0.05.

## Results

In rodent models, exposure to cannabinoids (both synthetic and plant-derived) during gestation or early development induces an array of deleterious consequences on behavior manifesting both at early life and adulthood (Hurd et al., 2019; Scheyer et al., 2019).

Perinatal exposure via lactation to either the plant-derived phytocannabinoid THC, or the synthetic cannabinoid, WIN, induces a significant delay in the trajectory of GABAergic development in the PFC of developing offspring, an effect which is accompanied by substantial behavioral alterations (Scheyer et al., 2020b). Further, the progeny of dams similarly exposed via lactation to THC during the first 10 d of postnatal life exhibit lasting deficits in synaptic plasticity in the PFC as well as augmented social behavior at adulthood (Scheyer et al., 2020a).

Here, we used this same protocol of perinatal cannabinoid exposure to determine whether synaptic and behavioral consequences are similarly produced following maternal administration of WIN. Thus, lactating dams were treated with a low dose of WIN (0.5 mg/kg, s.c.) or its vehicle (herein referred to as sham) from postnatal day (PND)1 to PND10. Experiments were then conducted in the male and female offspring at adulthood (>PND90).

All treatment effects (e.g., sham vs WIN) were found to be consistent across sexes. Thus, for figures and statistical analyses, data for male and female rats within treatment condition were combined. However, differences between the sexes within treatment conditions were noted in some measures. Details of within-treatment sex differences can be found in Tables 1–8.

**Table 1:** Social approach and social memory data by sex

Condition	Test – measure (unit)	Value	*t* test (M vs F)
Sham male	Social preference (ratio)	0.8647 ± 0.01743 *N* = 15	*p* = 0.5089
Sham female	Social preference (ratio)	0.8375 ± 0.03569 *N* = 8	
WIN male	Social preference (ratio)	0.9491 ± 0.01282 *N* = 11	*p* = 0.0255
WIN female	Social preference (ratio)	0.8618 ± 0.03219 *N* = 11	
Sham male	Social memory (ratio)	0.6147 ± 0.03886 *N* = 15	*p* = 0.0876
Sham female	Social memory (ratio)	0.7463 ± 0.05970 *N* = 8	
WIN male	Social memory (ratio)	0.6282 ± 0.05435 *N* = 11	*p* > 0.9999
WIN female	Social memory (ratio)	0.6282 ± 0.03590 *N* = 11	
Sham male	Social preference – time exploring object (s)	14.20 ± 1.853 *N* = 15	*p* = 0.8121
Sham female	Social preference – time exploring object (s)	13.61 ± 1.610 *N* = 8	
WIN male	Social preference – time exploring object (s)	6.586 ± 1.840 *N* = 11	*p* = 0.0420
WIN female	Social preference – time exploring object (s)	16.05 ± 3.822 *N* = 11	
Sham male	Social preference – time exploring rat (s)	95.38 ± 8.016 *N* = 15	*p* = 0.3569
Sham female	Social preference – time exploring rat (s)	83.02 ± 10.25 *N* = 8	
WIN male	Social preference – time exploring rat (s)	110.2 ± 9.669 *N* = 11	*p* = 0.4518
WIN female	Social preference – time exploring rat (s)	100.8 ± 7.422 *N* = 11	
Sham male	Social memory – time exploring familiar rat (s)	29.31 ± 3.733 *N* = 15	*p* = 0.2200
Sham female	Social memory – time exploring familiar rat (s)	20.90 ± 5.375 *N* = 8	
WIN male	Social memory – time exploring familiar rat (s)	26.17 ± 3.754 *N* = 11	*p* = 0.3621
WIN female	Social memory – time exploring familiar rat (s)	35.52 ± 2.748 *N* = 11	
Sham male	Social memory – time exploring novel rat (s)	47.06 ± 4.528 *N* = 15	*p* = 0.1116
Sham female	Social memory – time exploring novel rat (s)	58.58 ± 5.159 *N* = 8	
WIN male	Social memory – time exploring novel rat (s)	49.36 ± 8.196 *N* = 11	*p* = 0.7014
WIN female	Social memory – time exploring novel rat (s)	53.04 ± 4.657 *N* = 11	

### Perinatal exposure to a synthetic cannabimimetic alters social behavior and memory at adulthood

**Figure 1.**
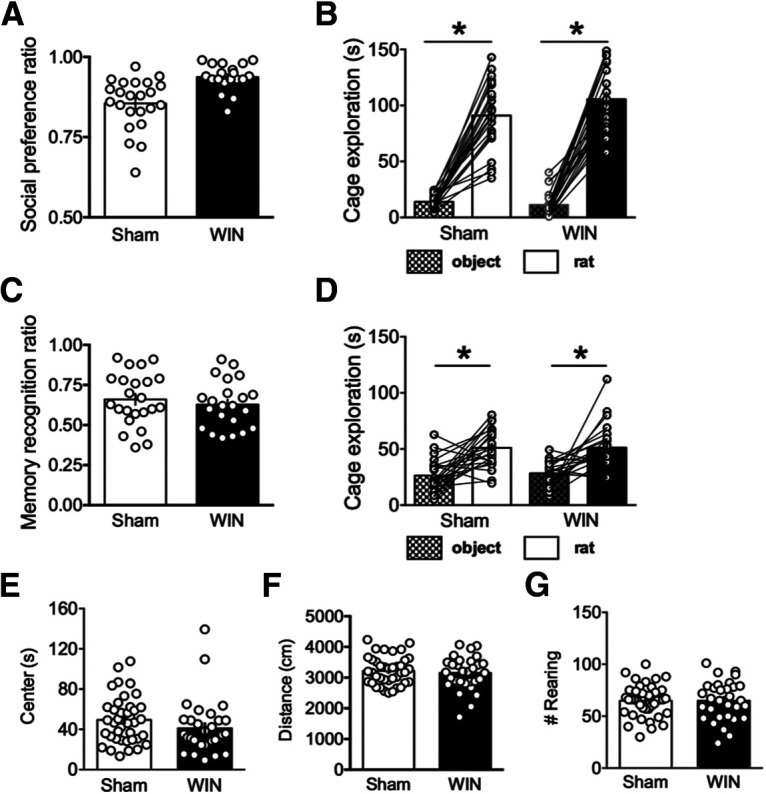
Perinatal WIN alters social approach, but not social memory behavior, nor behavior in the open field environment. ***A***, Adult offspring of WIN-treated dams exhibit significantly higher social preference than those of sham-treated dams (two-tailed *t* test, *p* = 0.0001; *N* = 23, 19, respectively). ***B***, Time spent exploring a novel rat is significantly higher than time spent exploring a novel object for both sham-exposed and WIN-exposed rats (one-way ANOVA, *F*_(3,86)_ = 14.49; Tukey’s *post hoc* analysis, *p* < 0.0001 for both groups; *N* = 23, 19, respectively). ***C***, ***D***, In the subsequent social memory test, the adult offspring of both sham-treated and WIN-treated dams exhibited significantly higher preference for a novel, as compared with familiar, rats. ***C***, The social memory index does not differ between the two groups (two-tailed *t* test, *p* = 0.557). ***D***, Time spent exploring a novel rat is significantly higher than time spent exploring a familiar rat for both sham-exposed and WIN-exposed rats (one-way ANOVA, *F*_(3,86)_ = 2.137; Tukey’s *post hoc* analysis, *p* < 0.0001 for both groups). ***E–G***, Behavior in the open field environment does not differ between the offspring of sham-treated and WIN-treated dams (*N* = 39, 31, respectively). Time spent in the left of the arena, total distance covered, and the number of rearing events is not significantly different between groups (two-tailed *t* tests, *p* = 0.1817, *p* = 0.5991, and *p* = 0.9783, respectively); *p < 0.05.

In order to determine whether the behavioral repertoire of WIN-exposed animals is altered at adulthood, we performed several behavioral analyses in both male and female rats. Because perinatally THC-exposed animals exhibited augmented social behavior at adulthood, we initiated a social approach and memory assay (Fig. 1*A–D*; Table 1).

First, during the social approach portion of the assay, WIN-exposed animals exhibited significantly heightened preference for a novel rat over a novel object, as compared with sham rats (Fig. 1*A*). Both sham-exposed and WIN-exposed rats exhibited a significant preference for the social stimulus as compared with the object (Fig. 1*B*). However, the magnitude of difference between time spent exploring the novel rat versus the novel object was significantly heightened in the adult offspring of WIN-treated dams. During the subsequent memory test, both sham-exposed and WIN-exposed rats exhibited a similar social preference for a novel rat over the familiar rat from the social approach assay (Fig. 1*C*,*D*).

Further, naturalistic behavior was observed before the social approach/memory testing by observing animals in the open field assay. No significant differences were noted in the time spent in the left of the arena, distance covered during the trial, or the exploratory behavior (number of rearing events) during the open field test (Fig. 1*E–G*; Table 2).

**Table 2:** Open field data by sex

Condition	Test – measure (unit)	Value	*t* test (M vs F)
Sham male	Open field – distance (cm)	3120 ± 80.10 *N* = 22	*p* = 0.1490
Sham female	Open field – distance (cm)	3348 ± 135.8 *N* = 17	
WIN male	Open field – distance (cm)	2861 ± 151.9 *N* = 14	*p* = 0.0070
WIN female	Open field – distance (cm)	3396 ± 97.26 *N* = 17	
Sham male	Open field – rearing (#)	62.23 ± 3.365 *N* = 22	*p* = 0.2443
Sham female	Open field – rearing (#)	68.18 ± 3.735 *N* = 17	
WIN male	Open field – rearing (#)	56.64 ± 5.840 *N* = 14	*p* = 0.0333
WIN female	Open field – rearing (#)	71.76 ± 3.118 *N* = 17	
Sham male	Open field – left (s)	54.85 ± 5.348 *N* = 22	*p* = 0.0839
Sham female	Open field – left (s)	42.24 ± 4.674 *N* = 17	
WIN male	Open field – left (s)	55.87 ± 8.835 *N* = 14	*p* = 0.0104
WIN female	Open field – left (s)	28.92 ± 2.733 *N* = 17	

### Perinatal exposure to WIN alters prefrontal synaptic plasticity at adulthood

**Figure 2.**
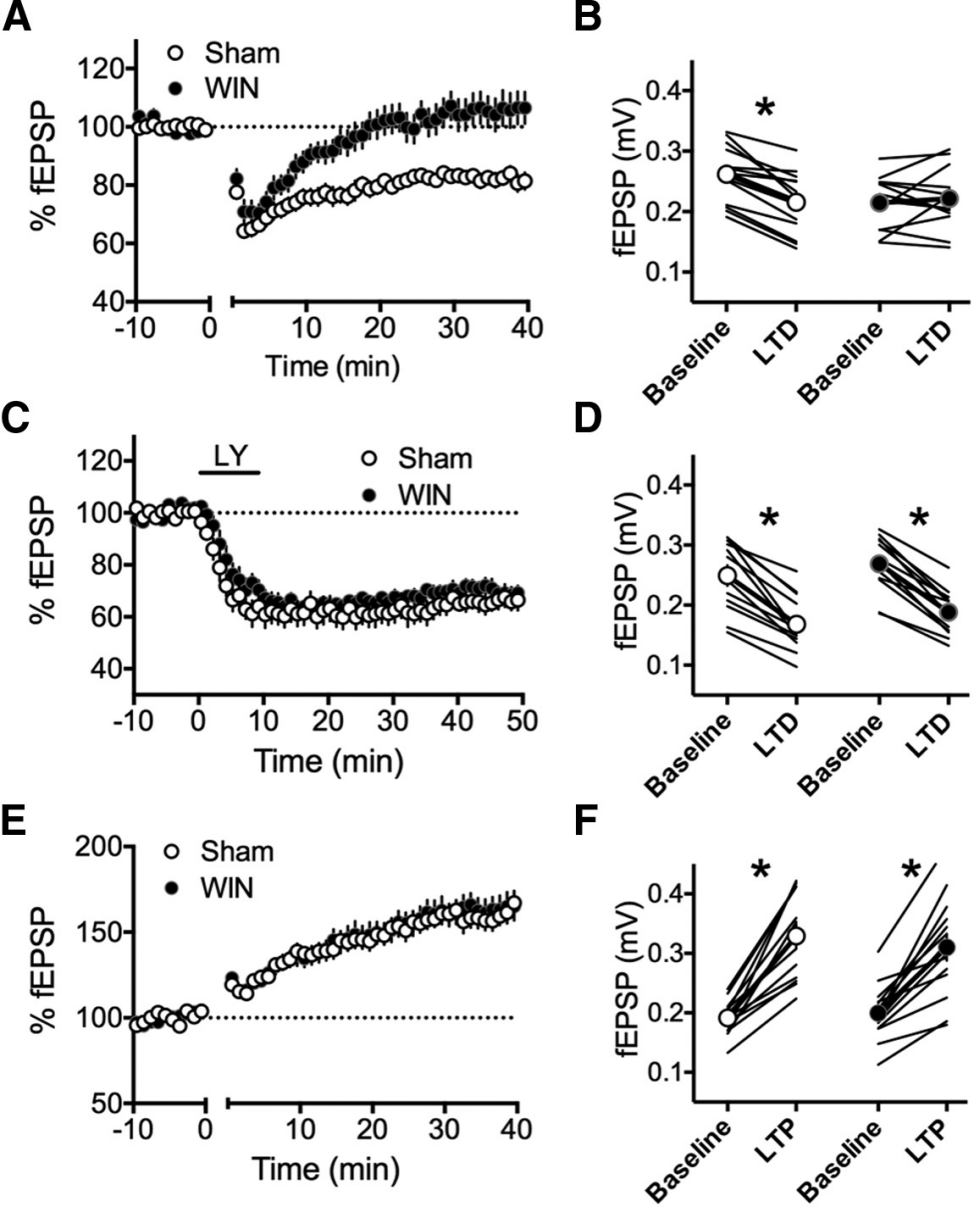
Perinatal WIN exposure induces a selective deficit in LTD in the PFC of adult offspring. ***A***, A 10-min, 10-Hz field stimulation of layer 2/3 cells in the PFC of the adult offspring of sham-treated dams (*N* = 14) elicited a robust eCB-LTD at deep layer synapses. However, this same protocol failed to induce eCB-LTD in the adult offspring of dams treat with WIN (*N* = 14). ***B***, fEPSP magnitude at baseline (−10−0 min) and LTD (35–40 min post-tetanus) values corresponding to the normalized values in ***A*** (two-way RM ANOVA, *F*_(1,24)_ = 16.58, *p* = 0.0004; Sidak’s multiple comparisons test, *p* = 0.0332 and *p* = 0.9412, respectively). ***C***, LTD mediated by mGlu_2/3_ receptors (mGluR-LTD) is not altered in WIN-exposed offspring. mGluR-LTD, induced via a 10-min application of LY379268 (LY; 30 nm), produced a significant depression at deep layer synapses of the PFC in the adult offspring of both sham-treated and WIN-treated dams (*N* = 12, 12, respectively). ***D***, fEPSP magnitude at baseline (−10−0 min) and LTD (30−40 min postdrug) values corresponding to the normalized values in ***C*.** No differences were found between groups comparing the 10-min baseline period and the last 10 min of recording, however both groups exhibited a significant difference of fEPSP magnitude at baseline (i.e., −10−0 min) as compared with 30−40 min postdrug (two-way RM ANOVA, *F*_(1,11)_ = 96.69, *p* < 0.0001; Sidak’s multiple comparisons test, *p* < 0.0001 for both groups). ***E***, A TBS protocol (five pulses at 100 hz, repeated four times) at layer 2/3 cells in the PFC of the adult offspring of both sham-treated and WIN-treated dams elicited a robust LTP at deep layer synapses (*N* = 13, 15, respectively). ***F***, fEPSP magnitude at baseline (−10−0 min) and LTP (30−40 min post-TBS) values corresponding to the normalized values in ***E*.** Both groups exhibited significant differences between the fEPSP magnitude at 30−40 min as compared with −10−0 min (two-way RM ANOVA, *F*_(1,25)_ = 1.737; Sidak’s multiple comparisons test, *p* < 0.0001 for both groups); *p < 0.05.

Perinatal THC exposure alters several forms of synaptic plasticity in the PFC at adulthood (Scheyer et al., 2020a). Thus, we elected to examine three forms of PFC plasticity to determine whether similar alterations followed perinatal WIN exposure. First, we used a 10 min, 10-Hz stimulation of superficial layers of the PFC to elicit an eCB-LTD at deep layer synapses (Fig. 2*A*,*B*; Table 3). Here, we found that while sham-exposed rats exhibited robust, lasting depression 30–40 min following the 10-min protocol, no such LTD was observed in the PFC of WIN-exposed rats. This finding is in line with our previous data showing an ablation of eCB-LTD in the PFC of THC-exposed rats.

**Table 3:** PFC LTD data by sex

Condition	Test – measure (unit)	Value	*t* test (M vs F)
Sham male	eCB-LTD – normalized fEPSP (35–40 min post-tetanus)	80.61 ± 3.408 *N* = 8	*p* = 0.5478
Sham female	eCB-LTD – normalized fEPSP (35–40 min post-tetanus)	83.90 ± 4.061 *N* = 6	
WIN male	eCB-LTD – normalized fEPSP (35–40 min post-tetanus)	103.9 ± 8.384 *N* = 6	*p* = 0.7042
WIN female	eCB-LTD – normalized fEPSP (35–40 min post-tetanus)	108.8 ± 9.488 *N* = 7	
Sham male	mGlu2/3-LTD – normalized fEPSP (35–40 min postdrug)	66.26 ± 4.196 *N* = 6	*p* = 0.8390
Sham female	mGlu2/3-LTD – normalized fEPSP (35–40 min postdrug)	67.58 ± 4.700 *N* = 6	
WIN male	mGlu2/3-LTD – normalized fEPSP (35–40 min postdrug)	67.48 ± 4.156 *N* = 7	*p* = 0.4467
WIN female	mGlu2/3-LTD – normalized fEPSP (35–40 min postdrug)	72.00 ± 3.900 *N* = 5	

Next, we examined a distinct form of LTD in the PFC mediated by mGlu2/3 receptors (Bara et al., 2018) which has previously been shown to be disrupted by chronic exposure to drugs (Hoffman et al., 2003; Huang et al., 2007; Kasanetz et al., 2013) and augmented at adulthood following perinatal THC exposure (Scheyer et al., 2020a). Thus, we exposed acute PFC slices to the mGlu2/3 agonist LY379268 (300 nm) to elicit an mGlu2/3-dependent LTD (Fig. 2*C*,*D*; Table 3). Here, we found that PFC synapses in slices obtained from the offspring of both sham-treated and WIN-treated dams exhibited a similar magnitude of mGlu2/3-dependent LTD at 30−40 min following drug application.

Finally, we used a θ-burst stimulation protocol at superficial layers of the PFC to induce a lasting synaptic potentiation (TBS-LTP) at deep layer synapses. Here, we found no alterations to the time course or magnitude of plasticity between slices obtained from the adult offspring of sham-treated, as compared with WIN-treated, dams (Fig. 2*E*,*F*; Table 4). Of note, these results stand in contrast to those from THC-exposed rats, wherein TBS-LTP is impaired at adulthood (Scheyer et al., 2020a).

**Table 4:** PFC LTP data by sex

Condition	Test – measure (unit)	Value	*t* test (M vs F)
Sham male	TBS-LTP – normalized fEPSP (35–40 min post-tetanus)	185.6 ± 26.32 *N* = 6	*p* = 0.3101
Sham female	TBS-LTP – normalized fEPSP (35–40 min post-tetanus)	155.4 ± 6.180 *N* = 7	
WIN male	TBS-LTP – normalized fEPSP (35–40 min post-tetanus)	168.0 ± 11.98 *N* = 9	*p* = 0.3917
WIN female	TBS-LTP – normalized fEPSP (35–40 min post-tetanus)	152.6 ± 12.58 *N* = 6	

**Figure 3.**
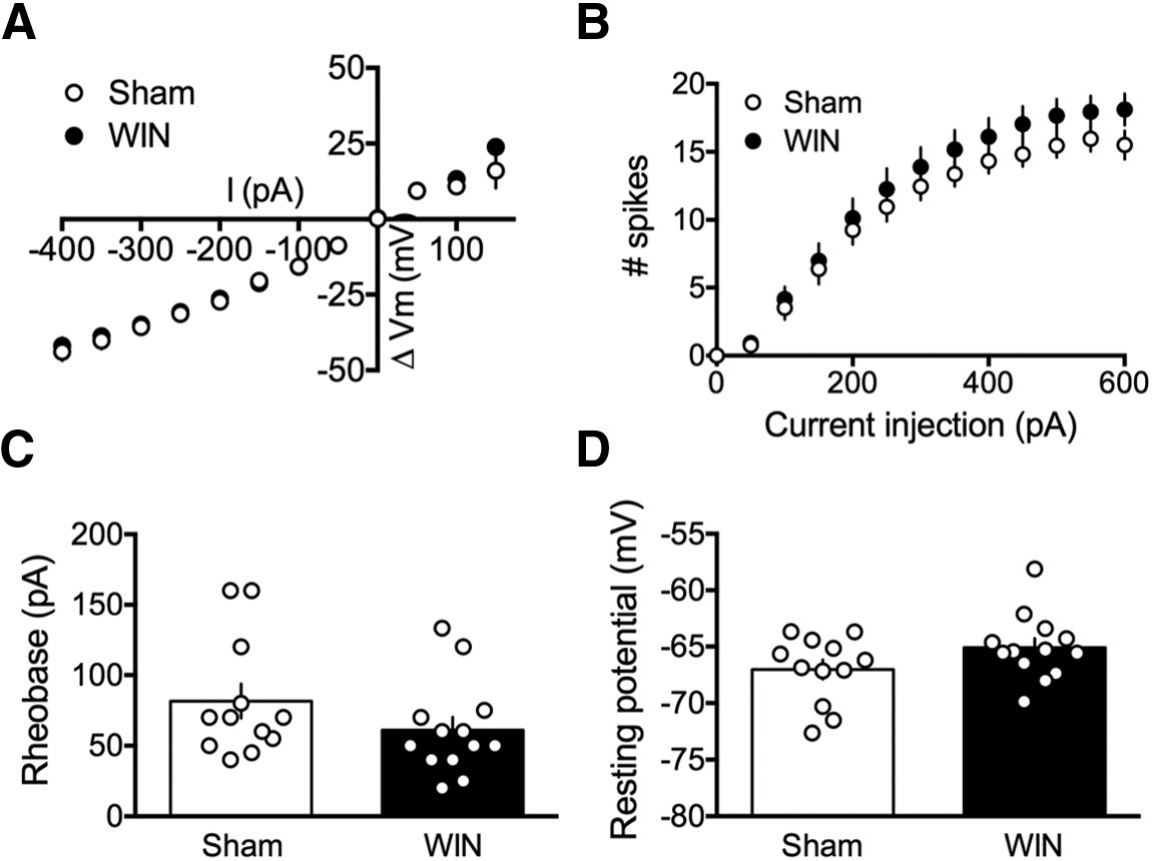
Perinatal WIN exposure does not alter properties of intrinsic excitability of deep layer pyramidal neurons in the PFC of adult offspring. ***A***, Current injection steps of 50 pA from –400 to 150 pA revealed no differences in the I-V relationship in pyramidal neurons of the PFC between the adult offspring of sham-treated and WIN-treated dams (*N* = 12, 13, respectively). ***B***, Action potentials elicited by progressive current injections from 0 to 600 pA revealed no difference in the number of spikes elicited in pyramidal neurons of the PFC in slices obtained from the adult offspring of WIN-injected dams as compared with those from sham-treated dams (*N* = 13, 12, respectively; two-way RM ANOVA, *F*_(20,460)_ = 1.112, *p* = 0.3328). ***C***, Progressive current injections in 10-pA steps from 0 to 200 pA revealed that the minimum current injection required to elicit an action potential (i.e., rheobase) did not differ in deep layer pyramidal neurons of PFC slices obtained from the adult offspring of WIN-treated, as compared with sham-treated, dams (*N* = 13, 12, respectively; two-tailed *t* test, *p* = 0.1896). ***D***, Similarly, no difference was found in the resting membrane potential of deep layer pyramidal cells in PFC slices obtained from the adult offspring of WIN-treated dams, as compared with those obtained from sham-treated dams (*N* = 13, 12, respectively; two-tailed *t* test, *p* = 0.1123); *p < 0.05.

Because perinatal THC exposure altered parameters of cell excitability in the PFC at adulthood, we next sought to determine whether WIN exposure elicited similar augmentations in excitability. Interestingly, pyramidal neurons in PFC slices obtained from the adult offspring of sham-treated and WIN-treated dams did not differ with regards to input–output excitability, spikes elicited by progressive current injections, nor in the rheobase or resting membrane potential (Fig. 3*A–D*; Table 5).

**Table 5:** NAc LTD data by sex

Condition	Test – measure (unit)	Value	*t* test (M vs F)
Sham male	TBS-LTP – normalized fEPSP (35–40 min post-tetanus)	82.37 ± 6.937 *N* = 5	*p* = 0.2893
Sham female	TBS-LTP – normalized fEPSP (35–40 min post-tetanus)	71.51 ± 6.589 *N* = 5	
WIN male	TBS-LTP – normalized fEPSP (35–40 min post-tetanus)	110.9 ± 6.257 *N* = 5	*p* = 0.0714
WIN female	TBS-LTP – normalized fEPSP (35–40 min post-tetanus)	95.53 ± 3.039 *N* = 4	

### Perinatal exposure to WIN alters synaptic plasticity and cellular properties in the accumbens at adulthood

**Figure 4.**
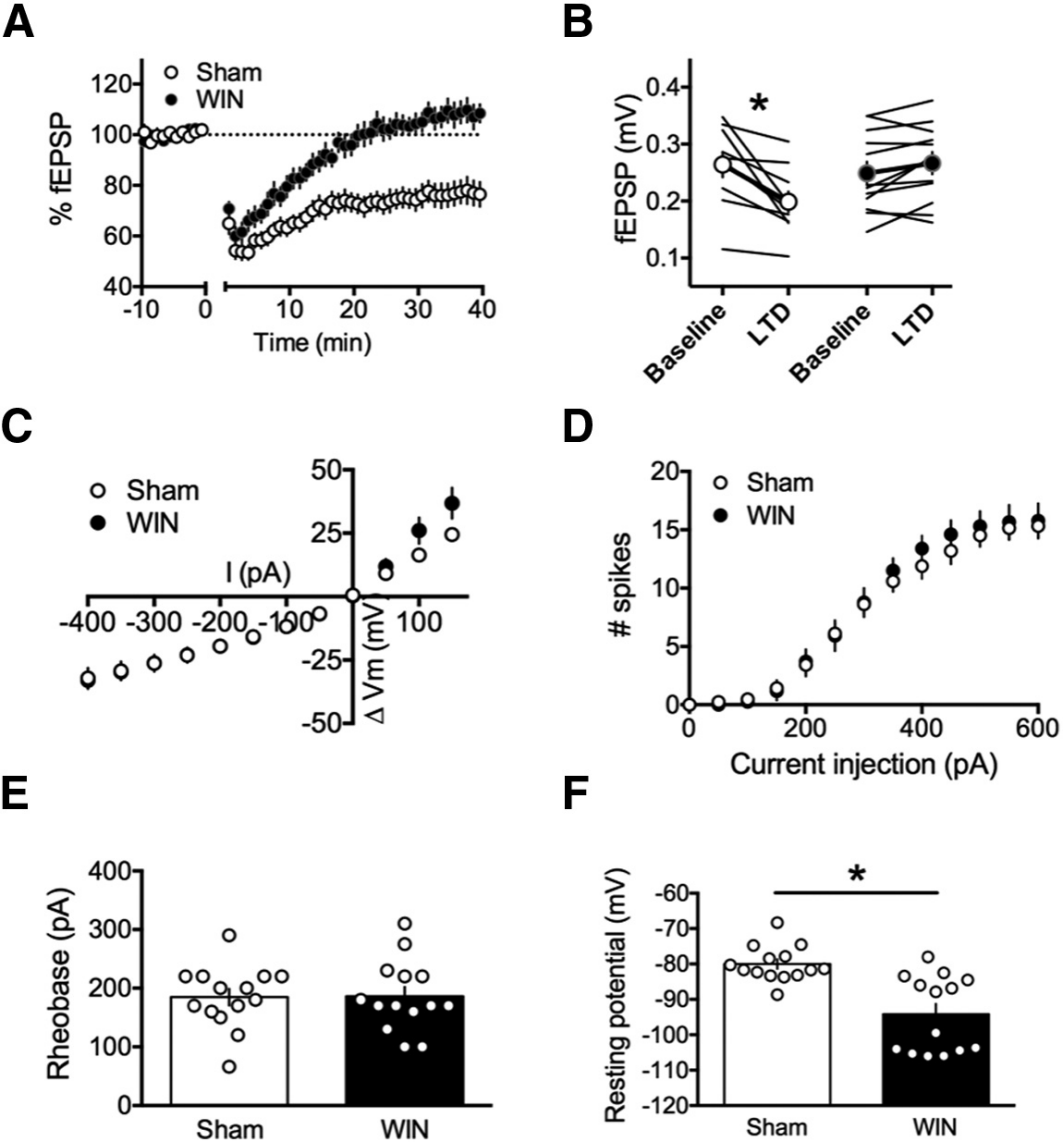
Perinatal WIN exposure abolishes LTD in the NAc of adult offspring and alters the resting membrane potential of NAc MSNs. ***A***, A 10-min, 10-Hz local field stimulation of the NAc of the adult offspring of sham-treated dams (*N* = 10) elicited a robust eCB-LTD. However, this same protocol failed to induce eCB-LTD in the adult offspring of dams treat with WIN (*N* = 12). ***B***, fEPSP magnitude at baseline (−10−0 min) and LTD (35–40 min post-tetanus) values corresponding to the normalized values in ***A*** (two-way RM ANOVA, *F*_(1,20)_ = 20.49, *p* = 0.0002; Sidak’s multiple comparisons test, *p* = 0.0002 and *p* = 0.3087 for sham and WIN, respectively). ***C***, Current injection steps of 50 pA from –400 to 150 pA revealed no differences in the I-V relationship in MSNs of the NAc between the adult offspring of sham-treated and WIN-treated dams (*N* = 14, 14, respectively). ***D***, Action potentials elicited by progressive current injections from 0 to 600 pA revealed no difference in the number of spikes elicited in pyramidal neurons of the PFC in slices obtained from the adult offspring of WIN-injected dams as compared with those from sham-treated dams (*N* = 14, 14, respectively; two-way RM ANOVA, *F*_(20,250)_ = 0.6092, *p* = 0.9071). ***E***, Progressive current injections in 10-pA steps from 0 to 200 pA revealed that the minimum current injection required to elicit an action potential (i.e., rheobase) did not differ in MSNs of NAc slices obtained from the adult offspring of WIN-treated, as compared with sham-treated, dams (*N* = 14, 14, respectively; two-tailed *t* test, *p* = 0.9502). ***F***, However, MSNs in NAc slices obtained from the adult offspring of WIN-treated dams exhibited significantly lower resting membrane potentials than those obtained from sham-exposed offspring (*N* = 14, 14, respectively; two-tailed *t* test, *p* = 0.0003); *p < 0.05.

Recent data have demonstrated that cannabinoids, experimenter-administered or self-administered, abolish LTD in the NAc (Mato et al., 2004, 2005; Neuhofer and Kalivas, 2018; Spencer et al., 2018). Thus, we sought to determine whether perinatal WIN exposure elicited similar deficits in LTD in the NAc at adulthood. We found that while the adult offspring of sham-treated dams exhibited robust LTD 30−40 min after a 10-min, 10-Hz stimulation, no such effect was found in the NAc of WIN-exposed rats (Fig. 4*A*,*B*; Table 6). Interestingly, unlike in the PFC, these alterations were accompanied by a significant reduction in the resting membrane potential of the principal neurons of the NAc, MSNs (Fig. 4*F*). No other parameters of cell excitability were found modified comparing MSNs in slices obtained from sham-exposed, as compared with WIN-exposed, rats (Fig. 4*C–E*; Table 7).

**Table 6:** PFC intrinsic properties data by sex

Condition	Test – measure (unit)	Value	*t* test (M vs F)
Sham male	Resting membrane potential (mV)	–67.95 ± 1.408 *N* = 6	*p* = 0.3580
Sham female	Resting membrane potential (mV)	–66.11 ± 0.9624 *N* = 6	
WIN male	Resting membrane potential (mV)	–64.12 ± 1.103 *N* = 7	*p* = 0.2000
WIN female	Resting membrane potential (mV)	–66.22 ± 1.077 *N* = 6	
Sham male	Rheobase (pA)	86,67 ± 18.06 *N* = 6	*p* = 0.7003
Sham female	Rheobase (pA)	76.67 ± 17.64 *N* = 6	
WIN male	Rheobase (pA)	66.19 ± 16.34 *N* = 7	*p* = 0.5487
WIN female	Rheobase (pA)	55.00 ± 7.303 *N* = 6	

**Table 7:** NAc intrinsic properties data by sex

Condition	Test – measure (unit)	Value	*t* test (M vs F)
Sham male	Resting membrane potential (mV)	–83.17 ± 1.463 *N* = 5	*p* = 0.0491
Sham female	Resting membrane potential (mV)	–78.26 ± 1.684 *N* = 9	
WIN male	Resting membrane potential (mV)	–93.95 ± 3.909 *N* = 8	*p* = 0.5281
WIN female	Resting membrane potential (mV)	–1004 ± 8.945 *N* = 6	
Sham male	Rheobase (pA)	199.2 ± 36.64 *N* = 5	*p* = 0.5827
Sham female	Rheobase (pA)	176.7 ± 10.93 *N* = 9	
WIN male	Rheobase (pA)	190.6 ± 20.19 *N* = 8	*p* = 0.7663
WIN female	Rheobase (pA)	180.0 ± 28.28 *N* = 6	

### Perinatal exposure to WIN enhances sucrose consumption at adulthood

**Figure 5.**
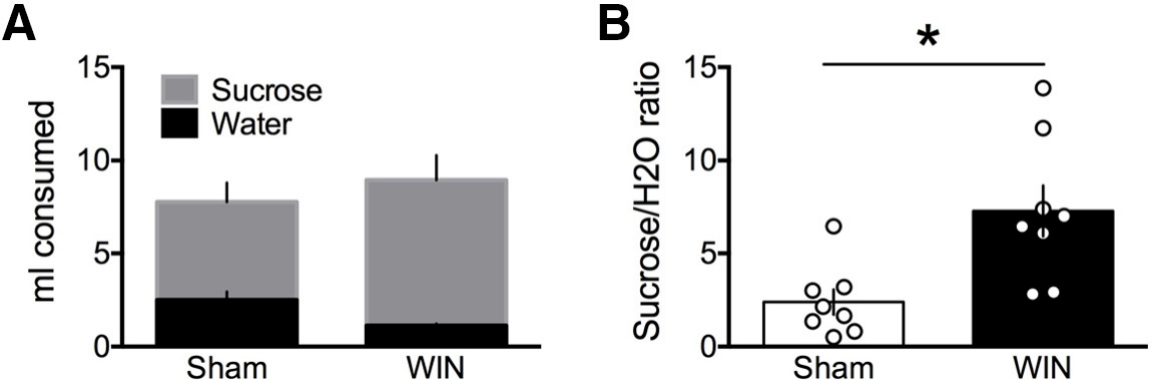
Perinatal WIN exposure increases sucrose preference in adult offspring. ***A***, Total quantities of water and 5% sucrose solution (ml) did not differ between the adult offspring of sham-treated or WIN-treated dams during a 20-min sucrose preference test (*N* = 8, 8, respectively). ***B***, Preference for the 5% sucrose solution over water was significantly higher in WIN- as compared with sham-treated rats (two-tailed *t* test, *p* = 0.0091). *p < 0.05.

The NAc plays an important role in reward-associated behavior, and recent data indicate a relationship between LTD in the NAc and reward-seeking behavior including sucrose consumption (Bobadilla et al., 2017; Bilbao et al., 2020). Thus, we examined the magnitude of sucrose preference in a two-bottle choice paradigm in the adult offspring of sham-treated and WIN-treated dams. Here, we found that while both groups exhibited a preference for a 5% sucrose solution (as compared with plain water) and consumed similar total quantities of liquid during the test (Fig. 5*A*; Table 8), the ratio of sucrose/water consumption was significantly higher in WIN- as compared with sham-treated adult offspring (Fig. 5*B*; Table 8). Thus, in addition to alterations to synaptic plasticity and intrinsic excitability of MSNs in the NAc, perinatal WIN exposure enhances reward-seeking behavior at adulthood.

**Table 8:** Sucrose preference data by sex

Condition	Test – measure (unit)	Value	*t* test (M vs F)
Sham male	Water consumed (ml)	1.425 ± 0.1181 *N* = 4	*p* = 0.0045
Sham female	Water consumed (ml)	3.575 ± 0.3326 *N* = 4	
WIN male	Water consumed (ml)	1.075 ± 0.2287 *N* = 4	*p* = 0.6953
WIN female	Water consumed (ml)	1.175 ± 0.04787 *N* = 4	
Sham male	Sucrose consumed (ml)	3.550 ± 1.533 *N* = 4	*p* = 0.1069
Sham female	Sucrose consumed (ml)	7.000 ± 0.7153 *N* = 4	
WIN male	Sucrose consumed (ml)	8.800 ± 1.564 *N* = 4	*p* = 0.5118
WIN female	Sucrose consumed (ml)	6.850 ± 2.291 *N* = 4	
Sham male	Sucrose preference (ratio)	2.739 ± 1.376 *N* = 4	*p* = 0.6530
Sham female	Sucrose preference (ratio)	2.039 ± 0.3597 *N* = 4	
WIN male	Sucrose preference (ratio)	8.674 ± 1.744 *N* = 4	*p* = 0.3462
WIN female	Sucrose preference (ratio)	5.885 ± 2.089 N = 4	

## Discussion

Here, using the synthetic cannabinoid WIN, we found that exposure to plant-derived phytocannabinoid THC via lactation induces behavioral and electrophysiological alterations lasting into adulthood. Specifically, we found altered social behavior, memory, and eCB-mediated synaptic plasticity in the PFC of adult offspring of dams administered WIN during the first 10 d of postnatal life. We also showing synaptic deficits and cellular alterations in the NAc along with enhanced sucrose preference, indicative of heightened reward seeking in WIN-exposed adults.

First, our behavioral analyses revealed that perinatal WIN exposure augments social preference in the adult offspring of WIN-treated dams. This result confirms the social augmentation seen following perinatal THC exposure (Scheyer et al., 2020a) but diverge from the effects of *in utero* THC exposure (i.e., social exploration was reduced). Such discrepancies point to potential differences in the sensitivity of developmental windows through the prenatal and early postnatal periods.

We also report that WIN exposure does not affect social memory, WIN abolishes novel object recognition at adulthood. Interestingly, social approach and memory is a complex behavior collating activity from diverse brain regions governing motivation and reward such as the amygdala (Adolphs, 2001) and NAc (Dölen et al., 2013). Indeed, augmentations in social approach behavior are often associated with decreased amygdalar function and signaling in the NAc, where oxytocin-mediated transmission is a key regulator of social approach and reward (Dölen et al., 2013) and is itself governed by the endocannabinoid system (ECS) (Wei et al., 2015). Previous data have highlighted the role of CCK interneuron dysfunction in WIN-mediated disruptions to social interaction (Vargish et al., 2017), a possible contributor to aberrant social behavior seen here that requires further investigation. Thus, variable impacts on memory and exploration behavior are likely attributable to underlying differences in the driving circuitry.

Results from the current study examining the long-term consequences of perinatal WIN exposure adds to a preliminary report of dysfunctional eCB-LTD in the PFC of perinatally THC-exposed offspring (Scheyer et al., 2020a). In contrast with THC treatment however, perinatal WIN did not lead to an enhanced magnitude of mGlu2/3-LTD nor a loss of TBS-LTP in the WIN-exposed progeny at adulthood. Differences in the pharmacokinetics, bioavailability, and pharmacological profiles of WIN and THC may explain these differences (Pertwee, 2005). Indeed, while WIN is a highly selective agonist of CB1, THC exhibits a diverse range of activity from partial agonist targeting of CB1 and CB2 to activation of several transient receptor potential channels, orphan receptors, and the nuclear PPARy. Despite these subtle differences, these data and those from previous studies suggest that alteration of PFC synaptic plasticity and social behavior at adulthood are common endophenotypes of perinatal cannabinoid exposure (Hoffman et al., 2003; Vargish et al., 2017; Bara et al., 2018; Scheyer et al., 2020a,b).

Perinatal THC exposure decreases excitability of principle neurons of the PFC (Scheyer et al., 2020a) in a fashion similar to chronic adolescent THC exposure in mice (Pickel et al., 2020). Here, we found that no such differences followed perinatal WIN exposure. These data point to a dissociation between measures of intrinsic excitability and synaptic plasticity within the PFC, as changes in these domains appear independent. Thus, alternative explanations for the loss of eCB-LTD must be considered in light of a lack of changes to cell excitability, including alterations to receptor function or other changes to the ECS such as alterations in eCB tone.

The NAc is essential to reward-associated behavior and eCB-mediated LTD in the NAc core controls reward-seeking behavior (Bilbao et al., 2020). Here, we found that this eCB-LTD is ablated in the NAc of the adult offspring of WIN-treated dams. This finding is in line with multiple reports of altered LTD in the NAc of cannabinoid-exposed animals (Mato et al., 2004, 2005; Neuhofer and Kalivas, 2018; Spencer et al., 2018). In contrast with our recordings in PFC principal neurons, we observed a significant reduction in the resting membrane potential of NAc MSNs. Further, in examining the reward-seeking behavior of these WIN-exposed offspring, we also found that the ratio of sucrose/water consumption in the two-bottle choice task was significantly higher in WIN-treated, as compared with sham-treated, adult offspring. Thus, in the NAc of WIN-exposed progeny, the loss of eCB-LTD and associated cell-excitability modifications were paralleled by modifications of reward-seeking behavior at adulthood.

In conclusion, these results indicate that perinatal exposure via lactation to a synthetic cannabinoid reproduces some of the long-lasting deficits induced at multiple scales by THC. Augmented social behavior and a loss of eCB-LTD in the PFC are therefore similar consequences of perinatal exposure to both naturally occurring phytocannabinoids and synthetic cannabimimetics. Additionally, we found that WIN exposure ablates eCB-LTD in the NAc, where the resting membrane potential of MSNs was found to be significantly decreased. These findings may indeed correlate with an enhanced sucrose-preference among WIN-exposed offspring. Together, these findings further illustrate the vulnerability of the developing brain and, consequently, behavior, to early-life insults to the endocannabinoid system via exposure to cannabinoid agonists.

## Synthesis

Reviewing Editor: Alfonso Araque, University of Minnesota Decisions are customarily a result of the Reviewing Editor and the peer reviewers coming together and discussing their recommendations until a consensus is reached. When revisions are invited, a fact-based synthesis statement explaining their decision and outlining what is needed to prepare a revision will be listed below. The following reviewer(s) agreed to reveal their identity: Carl Lupica, Ismael Galve-Roperh.

The reviewers have found merit and interest on the findings reported. I concur with the reviewers’ comments expressing the interest of the study. However, they also expressed some concerns about several issues that need to be addressed and clarified. I also consider pertinent the concerns expressed.

Reviewers and I believe that the comments (including additional experiments and analysis requested) might require less than two months of work.

I am looking forward to receiving a revised version of the manuscript with the reviewers´ comments addressed.

Comments of the reviewers:

Reviewer 1.

Advances the Field: The long-term effects of cannabis consumption during pregnancy and lactation are significant contemporary public health concerns. Therefore, this work is particularly relevant and timely. Moreover, most work in this field has been conducted using cannabis-derived phytocannabinoids. The present study examines effects of a synthetic cannabinoid, as these are also significant for public health.

Specific comments

In this manuscript the authors have explored the idea that exposure to psychoactive constituents of cannabis or synthetic cannabinoids during pregnancy and lactation periods disrupts the neuronal development of the prefrontal cortex and nucleus accumbens. This hypothesis is supported by prior studies showing that GABAergic neurons are particularly susceptible to this disruption by exposure to THC, the primary psychoactive component of cannabis, and with data indicating that behavior and synaptic physiological processes are altered in adulthood following this perinatal period exposure. Since the long-term effects of cannabis consumption during pregnancy and lactation are significant contemporary public health concerns, this work is particularly relevant and timely. The authors describe several changes in behavior and synaptic physiology in the adult offspring of rats exposed to the synthetic cannabinoid agonist WIN 55,212-2 during this perinatal period. They also compare these effects to previous work in which THC was given during the perinatal period. Interestingly, although many of the changes seen with WIN are similar to those observed with THC, there are several notable differences that, in my opinion, represent the more important findings in this study. I have made a few comments, detailed below, that are meant to help improve the manuscript.

1. Abstract, line 9. The authors state that “....perinatal programming of the gamma-aminobutyric acid trajectory in the prefrontal cortex....”. This is unnecessarily complex language to explain a basic observation; perinatal THC disrupts development of GABAergic neurons in these brain areas. Please clarify or qualify the statement by indicating the trajectory of GABA neuron development.

2. Fig 1B, you state that “Both Sham- and WIN-exposed rats did exhibit social preference, though the magnitude of difference between time spent exploring the novel rat versus the novel object was heightened in the adult offspring of WIN-treated dams.” However, it is unclear whether the difference in rat vs object was different between cannabinoid-exposed and sham groups. Was there a significant difference? This should be stated clearly.

3. Fig. 2a,b, it is unclear to what the black bars are referring in the WIN-exposed rats? To be consistent with the legend, shouldn’t this bar be white to indicate the response to a novel object2 in Fig 2a, and familiar object in Fig 2b? Also, the patterned bars should be the same for both groups to be consistent with these legends.

4. Fig 2b, and lines 240-242, and Discussion lines 315-325. How can rats have diminished memory for a novel object? It is novel. Therefore, they have not had experience with it, and there can be no memory. Is the deficit, perhaps in the motivation to interact with the novel object? As there are multiple possibilities that do not involve memory deficits to explain the reduced propensity to approach a novel object, these statements should be clarified throughout the manuscript.

5. Line 277, and 347-48. The first paper to show this is Hoffman et al., 2003, J Neurosci. 23:4815-20.

6. Line 306. Typo “showing"

7. The differences observed with the different forms of synaptic plasticity in the PFC between THC and WIN are among the most interesting observations in this work. The authors attribute this to potential differences in pharmacokinetics, bioavailability and pharmacological profiles of WIN and THC (line 331). However, no specifics are given. Moreover, there are additional possibilities that include involvement of non-cannabinoid receptors, as well as CB2Rs for the less specific agonist (THC). One way this could be addressed is to determine whether the effects of these drugs are prevented with selective antagonists for CB1Rs. However, this represents a large undertaking because a control group using antagonist alone would have to be included to compare with an agonist-antagonist co-treatment group. As an alternative, it would be helpful for the authors to address these possibilities using data that may be present in the literature. For example, are there other relevant published studies examining extended treatment with CB1 agonists and co-treatment with antagonists? It would be useful to discuss these possibilities and their relevance to unraveling the sites at which WIN and THC act perinatally to cause alterations in brain function in adulthood.

8. The authors begin the manuscript by explaining that perinatal cannabinoid exposure alters the trajectory of GABA neuron development in the PFC. However, there is no mention as to whether this is also noted in the nucleus accumbens, a brain region in which changes in synaptic plasticity was observed in the present study. Are there also developmental changes caused by perinatal exposure to cannabinoids in this brain structure? Also, along these lines, it is unclear how the changes in GABA neuron development caused by cannabinoids relate to the changes in synaptic plasticity outlined in the present work. This is especially puzzling because LTD involves changes at glutamate synapses and not GABA synapses. What, if any, is the connection between cannabinoid-induced changes in the trajectory of GABA neuron development and the changes observed in LTD in the PFC and nucleus accumbens?

Reviewer 2.

Advances the Field: The study aims to investigate the neurobiological consequences of gestational cannabinoid exposure. By using s.c. administration of the synthetic ligand WIN55,212-2 (PND1-10), the authors describe the existence of some behavioral changes and long-term synaptic plasticity adaptation in the PFC. The major drawback of the study is the use of a mixed synthetic cannabinoid ligand, WIN55,212-2, that has very different pharmacological properties than the main psychoactive compound of the cannabis plant THC. The differences between WIN55,212-2 and THC in parameters such as structure, solubility, affinity, potency, receptor selectivity and signaling bias cannot be defined as “subtle” (line 333). This is clear, for example, when differences in PFC synaptic plasticity are addressed. Whereas THC administration in the same developmental period augmented ECB-LTD and impaired theta burst stimulation-LTP, this was not observed when the WIN compound was used. Phytocannabinoids, namely THC, and not synthetic cannabinoid ligands constitute the real health problem for cannabis users that the authors attempt to model in their experimental setting.

In its present form the study provides a fragmented analysis and interpretation of the consequences of perinatal WIN exposure. The authors, for unknown reasons, have separated the data included in this study from a very similar THC-administration study, hence preventing the reader from a much more relevant characterization of the consequences of perinatal cannabinoid exposure. New experiments are required to clarify some noted inconsistencies with previous studies. Furthermore, the authors should substantially rewrite the Discussion to fairly analyze and interpret their findings considering the current knowledge of the field.

Comments:

Here authors describe the existence of non-sex dependent changes in social interaction (increased), novel object recognition (blunted), and sucrose preference (increased), while open field was unaffected. Considering that numerous studies, including some by the authors themselves, demonstrate that prenatal and perinatal THC or WIN administration exerts sex-dependent actions in neuronal development and behavior (Bara et al., 2018; de Salas et al., 2019; Manduca et al., 2020), the little attention paid to this aspect in the study is surprising indeed. Hence, it is necessary that the authors specify the sex-dependent differences they found (line 214, Tables 1-9) and illustrate them as appropriate.

In the study by Scheyer et al. (2020a), THC-induced social interaction was attributed to deficits in social discrimination, whereas here no changes in social memory are reported. Please clarify. On other forms of cognition, the authors describe decreased NOR (Fig. 2), which is hippocampal-dependent and is neglected along the study, as well as the important question of the nature of the neuronal populations affected by WIN administration and responsible of the functional consequences observed. Previous studies indicated that WIN interference with social interaction was mediated by CCK interneuronopaty (Vargish et al., 2016), and this also results in spatial cognitive impairment, but not in conceptual memory/NOR (de Salas et al., 2020). In addition, THC adolescent administration interferes with GABAergic PFC maturation and induce a mesolimbic hyperdopaminergic phenotype (LaViolettte studies, Frau et al.). The authors should correct the lack of inclusion of previous relevant work ignored in the manuscript, and put their findings taking within the frame of the whole current status of the field.

In a previous study, WIN administration for longer time periods (PND 5-20) induced alterations in ultrasonic vocalizations, homing behavior in a sex-dependent manner, but did not induce changes in temporal memory and anxiety (Manduca et al., 2020). The authors should reconcile these antecedents with the lack of differential sex-dependent sensitivity observed herein. In line with this, the authors should analyze the impact of perinatal WIN (and THC) administration in this modified administration paradigm (PND1-PND10) in ultrasonic vocalizations and social communication, homing behavior in order to elucidate whether different intensity of cannabinoid signaling exerts different consequences. This is a crucial aspect as developmental cannabinoid exposure has been shown to exert different neuronal plasticity and behavioral consequences when CB1 receptors are downregulated, as a consequence of agonist administration, or remain functionally active.

Technical note. In the NOR test, novel object 1 and 2 exploration times appear very segregated within each experimental group (Fig. 2a). Moreover a significant number of animals in both experimental groups do not show novel object preference, and only a few sham-treated animals demonstrate a significant novel object exploration time (Fig. 2b). These data suggest that the new objects are not well selected (Heyser and Chemero, 2012), hence raising concerns about the conclusion of the existence of NOR impairment.

Rearrange “Results” initial description and move it to Introduction or Methods section as appropriate.

## Author Response

Comments of the reviewers:

Reviewer 1.

Advances the Field: The long-term effects of cannabis consumption during pregnancy and lactation are significant contemporary public health concerns. Therefore, this work is particularly relevant and timely. Moreover, most work in this field has been conducted using cannabis-derived phytocannabinoids. The present study examines effects of a synthetic cannabinoid, as these are also significant for public health.

Specific comments

In this manuscript the authors have explored the idea that exposure to psychoactive constituents of cannabis or synthetic cannabinoids during pregnancy and lactation periods disrupts the neuronal development of the prefrontal cortex and nucleus accumbens. This hypothesis is supported by prior studies showing that GABAergic neurons are particularly susceptible to this disruption by exposure to THC, the primary psychoactive component of cannabis, and with data indicating that behavior and synaptic physiological processes are altered in adulthood following this perinatal period exposure. Since the long-term effects of cannabis consumption during pregnancy and lactation are significant contemporary public health concerns, this work is particularly relevant and timely. The authors describe several changes in behavior and synaptic physiology in the adult offspring of rats exposed to the synthetic cannabinoid agonist WIN 55,212-2 during this perinatal period. They also compare these effects to previous work in which THC was given during the perinatal period. Interestingly, although many of the changes seen with WIN are similar to those observed with THC, there are several notable differences that, in my opinion, represent the more important findings in this study. I have made a few comments, detailed below, that are meant to help improve the manuscript.

1. Abstract, line 9. The authors state that “....perinatal programming of the gamma-aminobutyric acid trajectory in the prefrontal cortex....”. This is unnecessarily complex language to explain a basic observation; perinatal THC disrupts development of GABAergic neurons in these brain areas. Please clarify or qualify the statement by indicating the trajectory of GABA neuron development.

We thank the reviewer for this comment and the suggestion of a simpler statement. We have integrated this suggestion in the Abstract.

2. Fig 1B, you state that “Both Sham- and WIN-exposed rats did exhibit social preference, though the magnitude of difference between time spent exploring the novel rat versus the novel object was heightened in the adult offspring of WIN-treated dams.” However, it is unclear whether the difference in rat vs object was different between cannabinoid-exposed and sham groups. Was there a significant difference? This should be stated clearly.

This description has been amended as follows: “Both Sham- and WIN-exposed rats exhibited a significant preference for the social stimulus as compared to the object (Figure 1b). However, the magnitude of difference between time spent exploring the novel rat versus the novel object was significantly heightened in the adult offspring of WIN-treated dams.”

We hope that this offers clarification.

3. Fig. 2a,b, it is unclear to what the black bars are referring in the WIN-exposed rats? To be consistent with the legend, shouldn’t this bar be white to indicate the response to a novel object2 in Fig 2a, and familiar object in Fig 2b? Also, the patterned bars should be the same for both groups to be consistent with these legends.

Thank you for identifying this potential confusion. This figure has been revised in accordance with your suggestions.

4. Fig 2b, and lines 240-242, and Discussion lines 315-325. How can rats have diminished memory for a novel object? It is novel. Therefore, they have not had experience with it, and there can be no memory. Is the deficit, perhaps in the motivation to interact with the novel object? As there are multiple possibilities that do not involve memory deficits to explain the reduced propensity to approach a novel object, these statements should be clarified throughout the manuscript.

Novel object recognition test failure indicates that rats did not exhibit a memory for the familiar object, thus approaching both the “familiar” and “novel” objects in part two of the test with a similar frequency. They do not display a memory for a novel object, they display a recognition of the novel object and a memory for the familiar object. We have amended the text and hope this is clearer, now.

5. Line 277, and 347-48. The first paper to show this is Hoffman et al., 2003, J Neurosci. 23:4815-20.

Thank you for alerting us to this oversight. This important paper has now been added to our references.

6. Line 306. Typo “showing"

Thank you for alerting us to this typo. The text has been corrected.

7. The differences observed with the different forms of synaptic plasticity in the PFC between THC and WIN are among the most interesting observations in this work. The authors attribute this to potential differences in pharmacokinetics, bioavailability and pharmacological profiles of WIN and THC (line 331). However, no specifics are given. Moreover, there are additional possibilities that include involvement of non-cannabinoid receptors, as well as CB2Rs for the less specific agonist (THC). One way this could be addressed is to determine whether the effects of these drugs are prevented with selective antagonists for CB1Rs. However, this represents a large undertaking because a control group using antagonist alone would have to be included to compare with an agonist-antagonist co-treatment group. As an alternative, it would be helpful for the authors to address these possibilities using data that may be present in the literature. For example, are there other relevant published studies examining extended treatment with CB1 agonists and co-treatment with antagonists? It would be useful to discuss these possibilities and their relevance to unraveling the sites at which WIN and THC act perinatally to cause alterations in brain function in adulthood.

We appreciate the insightful analysis of our data and the suggestion for additional experiments. As with the whole world of science, operations at our laboratory and animal facility are non-existent during the COVID-19 pandemic. Thus, we respectfully decline the suggestion of further experiments in this model, at this time. We have, however, performed unpublished experiments to address this question wherein either the dams or the offspring of WIN-treated dams were administered AM-251 in conjunction with WIN. Here, we observed no alterations to the developmental trajectory of GABAergic neurons in the PFC. Thus, we can confidently say that the changes seen here are resultant of CB1R activation. However, similar experiments have not yet been performed with THC-treated dams, and thus no comparison can be made.

Further discussion on the difficulties of determining the pharmacokinetics of subcutaneous WIN for apt comparison with THC can be found in Borsoi et al, 2019 (doi: 10.3389/fnbeh.2019.00023). Additionally, we have added to the discussion a brief comparison of WIN and THC receptor affinity profiles in order to further illustrate this point.

8. The authors begin the manuscript by explaining that perinatal cannabinoid exposure alters the trajectory of GABA neuron development in the PFC. However, there is no mention as to whether this is also noted in the nucleus accumbens, a brain region in which changes in synaptic plasticity was observed in the present study. Are there also developmental changes caused by perinatal exposure to cannabinoids in this brain structure? Also, along these lines, it is unclear how the changes in GABA neuron development caused by cannabinoids relate to the changes in synaptic plasticity outlined in the present work. This is especially puzzling because LTD involves changes at glutamate synapses and not GABA synapses. What, if any, is the connection between cannabinoid-induced changes in the trajectory of GABA neuron development and the changes observed in LTD in the PFC and nucleus accumbens?

We appreciate the insightful analysis of our data, here. As you note, the LTD seen in both the PFC and the NAc are glutamatergic, not GABAergic. However, developmental alterations to the excitatory/inhibitory balance at synapses remains a crucial factor in the functional valence of plasticity. As of yet, no reports have emerged tracking the development of GABAergic transmission in the NAc during early postnatal stages. Thus, we are unable to confirm or deny any correlation between the PFC developmental trajectory and that of the NAc. Mention of the disrupted PFC development seen with perinatal WIN or THC exposure was made in order to draw attention to the breadth of changes seen in the developing brain in our model of perinatal cannabinoid exposure, which, with these data, extend from P10 to >P100.

Reviewer 2.

Advances the Field: The study aims to investigate the neurobiological consequences of gestational cannabinoid exposure. By using s.c. administration of the synthetic ligand WIN55,212-2 (PND1-10), the authors describe the existence of some behavioral changes and long-term synaptic plasticity adaptation in the PFC. The major drawback of the study is the use of a mixed synthetic cannabinoid ligand, WIN55,212-2, that has very different pharmacological properties than the main psychoactive compound of the cannabis plant THC. The differences between WIN55,212-2 and THC in parameters such as structure, solubility, affinity, potency, receptor selectivity and signaling bias cannot be defined as “subtle” (line 333). This is clear, for example, when differences in PFC synaptic plasticity are addressed. Whereas THC administration in the same developmental period augmented ECB-LTD and impaired theta burst stimulation-LTP, this was not observed when the WIN compound was used. Phytocannabinoids, namely THC, and not synthetic cannabinoid ligands constitute the real health problem for cannabis users that the authors attempt to model in their experimental setting.

In its present form the study provides a fragmented analysis and interpretation of the consequences of perinatal WIN exposure. The authors, for unknown reasons, have separated the data included in this study from a very similar THC-administration study, hence preventing the reader from a much more relevant characterization of the consequences of perinatal cannabinoid exposure. New experiments are required to clarify some noted inconsistencies with previous studies. Furthermore, the authors should substantially rewrite the Discussion to fairly analyze and interpret their findings considering the current knowledge of the field.

Comments:

Here authors describe the existence of non-sex dependent changes in social interaction (increased), novel object recognition (blunted), and sucrose preference (increased), while open field was unaffected. Considering that numerous studies, including some by the authors themselves, demonstrate that prenatal and perinatal THC or WIN administration exerts sex-dependent actions in neuronal development and behavior (Bara et al., 2018; de Salas et al., 2019; Manduca et al., 2020), the little attention paid to this aspect in the study is surprising indeed. Hence, it is necessary that the authors specify the sex-dependent differences they found (line 214, Tables 1-9) and illustrate them as appropriate.

In the study by Scheyer et al. (2020a), THC-induced social interaction was attributed to deficits in social discrimination, whereas here no changes in social memory are reported. Please clarify. On other forms of cognition, the authors describe decreased NOR (Fig. 2), which is hippocampal-dependent and is neglected along the study, as well as the important question of the nature of the neuronal populations affected by WIN administration and responsible of the functional consequences observed. Previous studies indicated that WIN interference with social interaction was mediated by CCK interneuronopaty (Vargish et al., 2016), and this also results in spatial cognitive impairment, but not in conceptual memory/NOR (de Salas et al., 2020). In addition, THC adolescent administration interferes with GABAergic PFC maturation and induce a mesolimbic hyperdopaminergic phenotype (LaViolettte studies, Frau et al.). The authors should correct the lack of inclusion of previous relevant work ignored in the manuscript, and put their findings taking within the frame of the whole current status of the field.

We thank the reviewer for this insightful commentary. First, regarding the discrepancies between THC-included changes in social interaction and a lack of such changes seen here, we too are intrigued by this difference (indeed, it is one of the reasons we sought to publish these data). As noted in the manuscript, and now expanded upon at the behest of a different reviewer, THC and WIN do not share several properties including both pharmacokinetic and metabolic profiles as well as receptor interactions. Thus, assuming the two would produce similar results is not accurate despite their falling under the same banner of CB1 agonists.

Regarding your additional points, we have amended the manuscript to include some relevant associated findings in both the introduction and discussion sections.

In a previous study, WIN administration for longer time periods (PND 5-20) induced alterations in ultrasonic vocalizations, homing behavior in a sex-dependent manner, but did not induce changes in temporal memory and anxiety (Manduca et al., 2020). The authors should reconcile these antecedents with the lack of differential sex-dependent sensitivity observed herein. In line with this, the authors should analyze the impact of perinatal WIN (and THC) administration in this modified administration paradigm (PND1-PND10) in ultrasonic vocalizations and social communication, homing behavior in order to elucidate whether different intensity of cannabinoid signaling exerts different consequences. This is a crucial aspect as developmental cannabinoid exposure has been shown to exert different neuronal plasticity and behavioral consequences when CB1 receptors are downregulated, as a consequence of agonist administration, or remain functionally active.

We are glad the reviewer acknowledges Manduca et al. 2020. However, there seem to be an inaccuracy about the experimental protocol of this study. In this BJP paper the authors report the longitudinal analysis of multiple behavioral parameters in rats exposed in-utero (GD5-GD20). Thus, these rats were not exposed after birth PND 5-20 as suggested by the reviewer in this remark. We kindly refer the reviewer to our recently published study of the early life behavioral effects of WIN (PND 5-10) including homing and USV (Scheyer et al. Biol. Psy. 2019).

Technical note. In the NOR test, novel object 1 and 2 exploration times appear very segregated within each experimental group (Fig. 2a). Moreover a significant number of animals in both experimental groups do not show novel object preference, and only a few sham-treated animals demonstrate a significant novel object exploration time (Fig. 2b). These data suggest that the new objects are not well selected (Heyser and Chemero, 2012), hence raising concerns about the conclusion of the existence of NOR impairment.

We thank the author for this relevant technical note. We will seek in the future to replicate these experiments with additional objects in order to confirm or alter our conclusions.

Rearrange “Results” initial description and move it to Introduction or Methods section as appropriate.

We respectfully beg to differ. We prefer having a brief description of the study’s basis in the initial description in the results.
